# Closing the poor-rich gap in contraceptive use in urban Kenya: are family planning programs increasingly reaching the urban poor?

**DOI:** 10.1186/1475-9276-12-71

**Published:** 2013-08-27

**Authors:** Jean Christophe Fotso, Ilene S Speizer, Carol Mukiira, Paul Kizito, Vane Lumumba

**Affiliations:** 1 Innovations for MNCH, Concern Worldwide US, New York, NY, 10017, USA; 2 Independent Consultant, Population & Reproductive Health, Nairobi, Kenya; 3 Carolina Population Center, University of North Carolina at Chapel Hill (UNC), Chapel Hill, NC, 27599, USA; 4 African Population and Health Research Center (APHRC), P.O. Box 10787-00100, Nairobi, Kenya; 5 National Council for Population and Development (NCPD), Nairobi, Kenya

## Abstract

**Introduction:**

Kenya is characterized by high unmet need for family planning (FP) and high unplanned pregnancy, in a context of urban population explosion and increased urban poverty. It witnessed an improvement of its FP and reproductive health (RH) indicators in the recent past, after a period of stalled progress. The objectives of the paper are to: a) describe inequities in modern contraceptive use, types of methods used, and the main sources of contraceptives in urban Kenya; b) examine the extent to which differences in contraceptive use between the poor and the rich widened or shrank over time; and c) attempt to relate these findings to the FP programming context, with a focus on whether the services are increasingly reaching the urban poor.

**Methods:**

We use data from the 1993, 1998, 2003 and 2008/09 Kenya demographic and health survey. Bivariate analyses describe the patterns of modern contraceptive use and the types and sources of methods used, while multivariate logistic regression models assess how the gap between the poor and the rich varied over time. The quantitative analysis is complemented by a review on the major FP/RH programs carried out in Kenya.

**Results:**

There was a dramatic change in contraceptive use between 2003 and 2008/09 that resulted in virtually no gap between the poor and the rich in 2008/09, by contrast to the period 1993–1998 during which the improvement in contraceptive use did not significantly benefit the urban poor. Indeed, the late 1990s marked the realization by the Government of Kenya and its development partners, of the need to deliberately target the poor with family planning services. Most urban women use short-term and less effective methods, with the proportion of long-acting method users dropping by half during the review period. The proportion of private sector users also declined between 2003 and 2008/09.

**Conclusion:**

The narrowing gap in the recent past between the urban poor and the urban rich in the use of modern contraception is undoubtedly good news, which, coupled with the review of the family program context, suggests that family planning programs may be increasingly reaching the urban poor.

## Introduction

Family planning (FP) is now acknowledged as one of the most successful development interventions, with potential benefits on maternal and child health (MCH) outcomes, educational advances, economic development, and women’s empowerment [1]. Yet, 200 million women in the developing world want to delay pregnancy or stop childbearing, but are not using an effective method of contraception [2,3]. In developing countries as a whole, the proportion of married women using a method of contraception increased from 10% in the 1970s to nearly 60% in the late 1990s, while the total fertility rate (TFR) dropped from six children per woman to around three in the same period [2,4]. While Kenya followed a similar pattern of increased contraceptive use and substantial decline in fertility, unmet need for FP, which refers to the proportion of sexually active, fecund women who want to avoid or postpone childbearing but are not using any method of contraception [5], remains high at about 25% [6,7]. Noticeably, 42.7% of births in the five years preceding the 2008/09 Kenya Demographic and Health Survey (DHS) were reported to be unintended (25.2% mistimed and 17.5% unwanted), compared to 51.5% (34.9% mistimed and 16.6% unwanted) in 1993, representing a modest decline of just nine percentage points over a 15-year period [7]. According to the same study, the differences in unintended pregnancy by household wealth remained largely unchanged over time.

The consequences of low contraceptive use and high unmet need in terms of unintended pregnancies and births have been abundantly studied [2,8]. There is also ample evidence on the negative effects of unplanned pregnancy and fertility on infant, child, and mother’s health [2,6,9], household economic conditions, population growth, and the attainment of the Millennium Development Goals (MDGs) [10,11]. Equally well documented are the barriers to contraceptive uptake and the reasons for non-use [2,6,12,13].

Kenya, like most countries in sub-Saharan Africa, is experiencing an urban explosion. Its urban population made a great leap from about half a million in 1960 to about 2.5 million in 1980, further increased to reach about 9 million in 2010 (making up about 40% of the total population), and is projected to reach 40 million by 2050 [14]. While rural to urban migration is at play, evidence suggests that about 75% of urban growth in sub-Saharan Africa is due to natural population growth (difference between births and deaths), with only about 25% accounted for by migration to urban areas [15,16], pointing to the importance of access to FP services in urban areas, particularly among the urban poor.

Another dominant trend in Kenya’s population landscape is the growing urban poverty and deteriorated health outcomes among the urban poor. For example, over 60% of inhabitants of Kenya’s capital city, Nairobi, are estimated to be living in slums and other informal settlements [17], characterized by poor access to healthcare and reproductive health (RH) services, early sexual debut, and high-risk sexual behaviors [18,19]. Further, significant inequities in health and RH outcomes have been documented in urban Kenya, with the poor tending to have not only the lowest contraceptive use, but also the highest fertility and the highest unmet need for FP [18,20]. Admittedly, an important but largely neglected dimension of urban fertility dynamics is the reproductive outcomes of urban populations living in poverty [13]. In the new era of urban explosion and concomitant growth of urban slums and informal settlements, the urban population in general and the urban poor in particular, will increasingly play a dominant role in defining progress toward national and international development agendas, including the Millennium Development Goals (MDGs).

Within this background of high unmet need for FP in Kenya, high unplanned fertility, and urban population growth amidst increased urban poverty and deprivation, the objectives of the paper are three-fold. First, we describe inequities in modern contraceptive use, types of methods used, and the main sources of contraceptives in urban Kenya. Second, we examine the extent to which differences in contraceptive use between the better-off and less privileged groups widened or shrank over time. Third and finally, using a review of major FP programs implemented in the country, we attempt to relate these findings to the FP programming context, with a focus on whether the services are increasingly reaching the urban poor.

### Population and family planning in Kenya: policy context and major achievements

Kenya is recognized for its robust population, FP and RH policy environment. It was one of the first countries in sub-Saharan Africa to develop a national population policy and launch a national FP program in the late 1960s; these policies laid the foundation for the onset of the Kenya demographic transition in the 1980s [21,22]. During the 1980s and early 1990s, the government continued to demonstrate considerable commitment to FP, with the development of new national policies and guidelines which paved the way for increased support for contraceptive supplies and extensive information, education and communication (IEC) campaigns [22,23]. Service provision expanded impressively during the period (80s to early 90s), while at the same time, the country registered a rapid decline in fertility – from an average of 8.1 children per woman in 1977 to 4.7 in 1998, and a steady rise in the modern contraceptive prevalence rate (CPR) from 7% in 1977 to 39% in 1998 [7,24].

In the mid-1990s the national FP program started to dwindle, and external funding for FP services and IEC declined, as other competing priorities gained traction in the global and national agendas (particularly the HIV/AIDS pandemic). The positive trends described above came to a halt in the late 1990s, with stalled contraceptive prevalence and fertility decline [21,24,25]. The 2004 Kenya Service Provision Assessment Survey found that in the five years preceding the survey, the proportion of health facilities offering any method of FP declined from 88% to 75% [26].

From the mid-2000s, champions of FP/RH within the Kenyan Government began to play important roles in refocusing energies on FP through policy and advocacy activities, providing a framework for equitable, efficient, and effective delivery of high-quality RH services throughout the country, and emphasizing reaching those in greatest need and most vulnerable [26,27]. A line item for contraceptive commodities was eventually included in the 2005 national budget, allocating 200 million Kenyan Shillings (or US$2.62 million), increasing to 300 million Kenyan shillings in the 2006/7 financial year [21,23]. The latest data indicate that contraceptive use once again registered an increase in 2008/09 – to 46% from 39% in 2003 - while fertility declined modestly to 4.6 births per woman, down from 4.9 in 2003, returning just below its 1998 level of 4.7 [7]. While these national trends in FP use and fertility are essential to assess progress towards the MDGs, understanding the urban dynamics and the vulnerabilities of the urban poor, is of special interest, as the country becomes increasingly urban.

## Data and methods

This study uses secondary data from the urban samples of the 1993, 1998, 2003 and 2008/9 Kenya Demographic and Health Survey (DHS). As in other countries, the surveys are household-based, and designed to allow representative samples for urban and rural areas separately. The surveys utilized a two-stage sample design, with sample clusters selected in the first stage, and households selected in the second stage. The DHS individual women’s questionnaire asks a set of questions about contraceptive use and source of the contraceptive methods. We restrict the sample to currently married women as this is the standard sample used to measure contraceptive use, unmet need, and the MDGs. The quantitative analysis is complemented by a review on the major FP programs carried out in Kenya between the late 1990s and the late 2000s. The search was mainly directed towards grey and unpublished literature, with attention to documents and reports available from the websites of Kenya’s major development partners. Combinations of the following keywords were used: family planning, reproductive health, Kenya, urban, and urban poor. Bibliographies of relevant papers were also used as additional sources of information.

### Variables

The key dependent variable for this analysis is use of modern contraceptive methods. In the DHS, women are asked: “Are you currently doing something or using any method to delay or avoid getting pregnant?” If yes, they are asked which method they are currently using. Women who report using two methods are coded based on the most effective method reported. Modern methods reported in the Kenya DHS include pills, injections, female and male condoms, intrauterine device (IUD), spermicides, implant, female and male sterilization, and lactational amenorrhea (LAM). These methods are further classified as short-term (pills, injections, spermicides, female and male condoms, and LAM), long-term (IUD and implant), and permanent (female and male sterilization). Traditional methods (periodic abstinence, withdrawal and other folkloric methods) were not included in the study. Not only are they less effective and reliable, our exploratory analysis showed that they remained marginal, varying between 5.5% and 7.5% during the period under review, with very minimal differences by wealth or education. Equally of interest to this paper is the source of contraceptives, recoded as public or private/other (including non-governmental and faith-based organizations).

The main predictor is poverty status. In this paper, as in most studies using DHS data, in the absence of data on income or expenditures, we use the household wealth index [28], recalculated on the urban sample. The wealth index is constructed from household’s assets using principal components analysis (PCA). Asset variables available in datasets include source of drinking water; type of toilet facility; main material of floor, wall and roof; presence of electricity, and ownership of durable goods like radio, television, refrigerator, bicycle and phones. The variable generated is recoded as tertiles (i.e. three categories of equal size), with categories labelled poor, middle, and rich. The wealth index was constructed for each year using available assets included in the study year. At the exploratory stage, we attempted to construct an overall wealth index on the pooled data. This approach proved challenging due to the change across the years in the number, type and coding of assets. Moreover, the role of varying assets to measure wealth changed markedly over the years (e.g. access to cell phones changed dramatically over the 15-year study period).

Measurement and meaning of health inequities remain a subject of debate in the public health literature [29]. The notion of relative index of inequality (RII) and slope index of inequality (SII) have gained attention in the recent past [30], providing additional insights into health inequalities beside other long-standing measures such as the concentration index and the frequency ratio between the uppermost and lowermost categories of a polytomous socioeconomic variable [30]. In this paper, we use the PCA weighted wealth index described above as this approach is most often used with DHS data and lends itself to easier interpretation by program implementers and policy makers. We complement this approach with the effects of education, another domain of inequity (see description below).

Women’s education is also used as a secondary dimension of poverty. The variable is coded as none, primary, and secondary or higher. The multivariate analysis on trends and equities in modern contraceptive use controls for religion (Catholic, other Christian, Muslim/others), region (the country’s eight provinces), women’s age (below 25, 25–34, and 35 or older), number of living children (0–1, 2–3, 4+), and fertility intention (wants a child within two years, wants a child after two years or is unsure or undecided, wants no more children or is sterilized or infecund). To reduce selection bias, we included the 64 women (out of 4,306) who reported being infecund in the wants no more children/sterilized category. Because some of these women were users of modern contraception, dropping them would have biased the analysis of modern method use.

### Statistical methods

Weighted bi-variate analyses are used to describe the patterns of modern contraceptive use and the types and sources of methods used, with the strength of the associations measured by chi-square tests. Multivariate logistic regression models are employed to estimate the crude and adjusted effects on contraceptive use of the predictors of interest, and in particular to assess how the gap between the poor and the rich varied over time. Analyses take into account the hierarchical structure of the data as a result of the sampling design, with women clustered within households, and households, in turn, nested within sampling clusters [31]. Because the number of women per household is small, we collapse the household and women levels and run two-level (sampling clusters and women/households) logistic regression in three stages, with modern contraceptive use as the dependent variable: Model 1 includes all three predictors and all five control variables. To assess the extent to which the poor-rich difference in contraceptive use increased or decreased over time, Model 2 adds to Model 1 an interaction between household wealth and survey year. Similarly, Model 3 adds to Model 1 an interaction between education and survey year. The STATA command *xtlogit* is used.

## Results

### Sample characteristics

Table 1 describes the sample of Kenyan urban married women from the 1993, 1998, 2003, and 2008/09 DHS. Although the percentage of women with primary education remained largely unchanged over time (at around 43%), that of women with secondary or higher education rose markedly from about 47% in 1993 to 57% in 2008/09. The percentage of women with no education dropped correspondingly from 10% to 5% during the same period. Table 1 also demonstrates that a majority of women are Catholic (22%) or of another Christian religion (62%), are between 25 and 34 years old (44%), have 2–3 children (43%), or want to stop childbearing/are sterilized/infecund (48%). Changes over time are noted in the steady decline of the proportion of women with four or more children, despite the increase in the proportion of women aged 35 years or older, corroborating the fertility decline in urban Kenya. Finally, after a period of stalled increase between 1998 and 2003 at around 40%, modern contraceptive use rose in urban Kenya to nearly 47% in 2008/09.

**Table 1:** **Characteristics**^**1 **^**of currently married women: Urban Kenya - 1993, 1998, 2003 and 2008/09**

	**1993**	**1998**	**2003**	**2008/09**	**All four surveys**
Household wealth^2^					
Poor	28.6	28.3	31.3	31.6	30.1
Middle	31.8	33.9	34.5	34.8	34.0
Rich	39.6	37.9	34.2	33.6	35.9
Women education					
No education	10.2	5.3	8.1	5.1	6.9
Primary	43.2	44.4	44.1	38.3	42.3
Secondary+	46.6	50.3	47.8	56.6	50.8
Religion					
Catholic	26.4	27.1	21.1	15.5	21.9
Other Christian	54.4	56.0	64.4	68.2	61.6
Others	19.2	16.8	14.5	16.4	16.5
Region					
Nairobi	38.9	40.4	38.3	31.5	36.9
Central	6.5	4.3	8.1	6.9	6.5
Coast	15.7	15.5	14.7	19.5	16.5
Eastern	6.5	7.2	4.7	6.8	6.3
Nyanza	5.6	13.9	7.9	8.0	9.1
Rift Valley	18.5	13.4	20.1	20.2	18.1
Western	8.5	5.2	4.2	5.6	5.6
North Eastern	NA	NA	2.1	1.5	1.0
Woman’s age					
<24	31.5	31.5	29.8	24.9	29.1
25-34	46.8	42.8	41.7	45.7	44.0
35+	21.6	25.8	28.6	29.4	26.9
Number of living children					
0-1	34.1	32.7	35.7	35.2	34.5
2-3	38.7	42.8	40.8	46.0	42.5
4+	27.2	24.5	23.5	18.8	23.0
Fertility intentions					
Want a child within 2 years	15.4	17.6	21.4	17.4	18.2
Wants a child after 2 years/unsure/undecided	34.3	31.1	32.3	38.2	34.1
Wants no more/sterilized/infecund	50.3	51.3	46.3	44.4	47.7
Modern contraceptive use	37.9	41.0	39.9	46.6	41.8
N	607	838	1,440	1,421	4,306

^1^Weighted percentages.

^2^The wealth variable is constructed at the household level (for each urban sample). Women are thus not evenly distributed in the three wealth groups.

### Modern contraceptive use: are inequities narrowing or widening?

**Figure 1:**
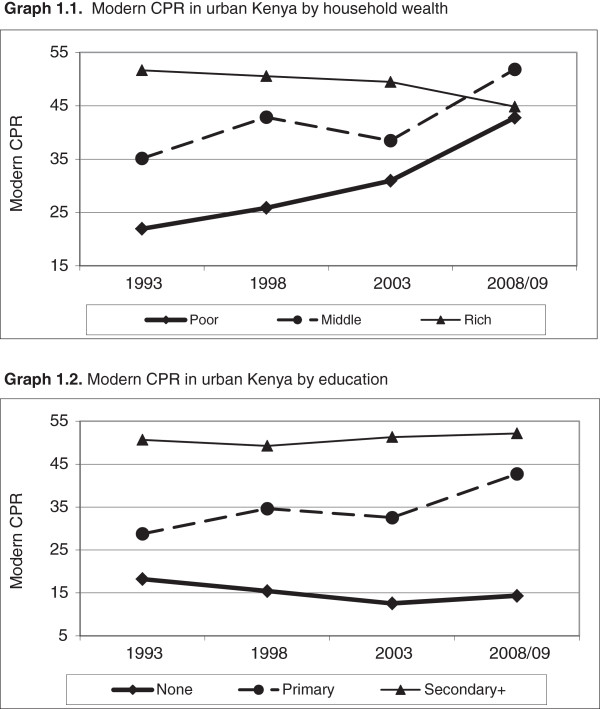
Inequities in contraceptive prevalence rate: Urban Kenya - 1993, 1998, 2003 and 2008/09.

Figure 1 shows the trends and socio-economic differentials in the modern CPR by household wealth (Graph 1.1) and women’s education (Graph 1.2). As can be seen, Graph 1.1 shows a pattern of slow narrowing of the poor-rich inequities in the use of modern FP methods between 1993 and 2003, with modern CPR increasing from 22.0% to 31.0% among the urban poor, and stalling around 50% among the urban rich. As a result, urban rich women were about 2.4 times (51.7% versus 22.0%) more likely than the urban poor to use modern contraception in 1993; the ratio dropped to 2.0 (50.6% versus 25.9%) in 1998, and to 1.6 (49.5% versus 31.0%) in 2003. During 2003 and 2008/09, there was an abrupt change that resulted in virtually no difference between the urban poor and the urban rich in the use of modern methods of contraceptives in 2008/09. Modern CPR increased by nearly twelve percentage points among women in the lowest tertile (the poor), and declined by five percentage points among women in the highest tertile (the rich), resulting in two percentage points difference between the poor and the rich in 2008/09 (CPR of 42.8% among the poor and 44.9% among the rich).

Graph 1.2 also depicts a declining contraceptive use difference between urban women with primary education and their counterparts with secondary or higher education over time. While in 1993, women with secondary education were about 80% more likely to use a modern method of contraception than their peers with primary education (CPR of 50.7% versus 28.8%), the gap dwindled to around 1.5 in 1998 and 2003, and further dropped to 1.2 in 2008/09 (CPR of 52.2% versus 42.7%). This pattern is a result of a stalled trend among women with secondary or higher education (at around 51%) and a steep increase among those with primary education (from 29% in 1993 to 42.7% in 2008/09). There has been little change in modern FP use among women with no education. They represent less than seven percent of the sample, hence the focus on the two other educational groups.

Table 2 presents the multivariate analysis of trends and inequities in modern contraceptive use in urban Kenya. Model 1 confirms a statistically significant CPR increase between 2003 and 2008/09 (p < 0.01), and large wealth and education disparities in the use of modern contraception (p < 0.001). Model 2 presents the interaction between household wealth and survey year. The statistically significant and negative coefficient of the interaction term “Rich-2008/09” (−0.529, p < 0.05) indicates that the poor-rich gap in 2008/09 was almost negligible (coefficient of 0.533-0.529 = 0.004), down from a statistically significant poor-rich difference in 2003, as illustrated by the coefficient of 0.533 (p < 0.01). The results in Model 3, which shows the interaction between women’s education and survey year, are similar to those in Model 2, although there remains a significant difference between the highest education and the lowest in 2008/09. There are strong educational differences in modern CPR in 2003 (p < 0.001), and a negative and statistically significant term for “Secondary + − 2008/09” (−0.338, p < 0.05), indicating a drop in the education gap between 2003 and 2008/09 (coefficient of 0.720-0.382 = 0.382).

**Table 2:** Logistic regression models (coefficients and p-value) on trends and inequities in modern contraceptive use: Urban Kenya - 1993, 1998, 2003 and 2008/09

	**Model 1**			**Model 2**			**Model 3**		
	**Coefficient**	**P-value**		**Coefficient**	**P-value**		**Coefficient**	**P-value**	
Survey year [Ref: 2003]									
1993	−0.155	0.217		−0.438	0.069	†	−0.152	0.407	
1998	−0.044	0.703		−0.101	0.624		0.166	0.306	
2008/09	0.275	0.005	**	0.487	0.003	**	0.474	0.001	**
Household wealth [Ref: Poor]									
Middle	0.414	0.000	***	0.378	0.020	*			
Rich	0.446	0.000	***	0.533	0.002	**			
Woman’s education [Ref: Primary]									
None	−0.774	0.000	***				−0.637	0.030	*
Secondary +	0.529	0.000	***				0.720	0.000	***
Interaction Wealth-Survey time									
Middle-1993				0.274	0.376				
Middle-1998				0.162	0.541				
Middle-2008/09				−0.096	0.661				
Rich-1993				0.449	0.142				
Rich-1998				0.006	0.983				
**Rich-2008/09**				**−0.529**	**0.018**	*			
Interaction Education-Survey time									
None-1993							0.011	0.981	
None-1998							−0.175	0.697	
None-2008/09							−0.345	0.406	
Secondary + −1993							0.002	0.994	
Secondary + −1998							−0.385	0.046	*
**Secondary + −2008/09**							**−0.338**	**0.042**	*

†p < .10; *p < .05; **p < .01; ***p < .001.

Model 1 controls for religion, region (province), and women’s age, number of living children and fertility preferences (coefficients not shown).

Model 2 adds the interaction of household wealth and survey time to Model 1 (Coefficients of interest are in bold).

Model 3 adds the interaction of woman’s education and survey time to Model 1 (Coefficients of interest are in bold).

### Types and source of methods: differences by economic status

Table 3 presents the distribution of urban married women currently using a modern method of contraception by the type of method defined as short-term methods and long-acting/permanent methods (LAPMs). About 72% of current users on average across the four surveys resorted to short-term methods (pills, injections and condoms). More worrying is the sharp decline over time of the proportion of LAPMs users (from 39% in 1993 to nearly 18% in 2008/09), and the corresponding increase in the proportion of users of short-term methods (from about 61% to 82%). As expected, rich or more educated women are more likely to use a LAPM, compared to their poorer or less-educated counterparts. Table 3 also suggests that the gap in use of LAPM between the more educated and the less educated has tended to widen over the years, especially between 2003 and 2008/09. The disparities between the poor and the rich on the other hand, narrowed between 1993 and 2003, but increased markedly afterwards.

**Table 3:** **Types and sources of modern contraceptive methods used, by wealth and education**^**1**^**: Urban Kenya - 1993, 1998, 2003 and 2008/09**

	**1993**	**1998**	**2003**	**2008/09**	**All surveys**
**1. Type of methods used**					
Short term^2^	61.0	66.4	72.7	81.7	72.2
Long acting^3^	25.2	19.2	17.0	11.9	17.2
Permanent^4^	13.8	14.4	10.4	6.5	10.7
Use of long acting and permanent methods by Household wealth^5^					
Poor	23.7	26.2	25.5	13.6	20.5
Middle	38.8	24.2	21.4	11.7	20.9
Rich	43.9	43.5	33.0	30.6	37.6
p-value^6^	0.131	0.012	0.004	0.000	0.000
Rich/poor ratio	1.8	1.7	1.3	2.3	1.8
Use of long acting and permanent methods by education					
None/Primary^7^	35.8	25.6	20.1	11.8	21.4
Secondary+	41.0	38.8	31.8	22.1	31.8
p-value	0.566	0.035	0.003	0.000	0.000
Secondary+/primary ratio	1.1	1.5	1.6	1.9	1.5
**2. Source of method used**^**8**^					
Public	56.5	52.6	44.9	49.2	50.1
Private/other	43.5	47.4	55.1	50.8	49.9
Use of private/other sources by Household wealth					
Poor	30.0	45.6	34.3	31.7	34.1
Middle	23.2	34.8	47.1	51.5	42.3
Rich	60.9	57.7	73.4	67.1	64.9
p-value	0.000	0.000	0.000	0.000	0.000
Rich/poor ratio	2.0	1.3	2.1	2.1	1.9
Use of private/other sources by education					
None/Primary	39.9	42.4	38.0	45.8	41.9
Secondary+	45.7	50.7	65.9	53.7	54.9
p-value	0.435	0.330	0.000	0.034	0.000
Secondary+/primary ratio	1.1	1.2	1.7	1.2	1.3
N (un-weighted)	222	318	550	626	1,716

^1^Weighted percentages.

^2^Short-term methods: pills, injections, spermicides, condoms and lactational amenorrhea.

^3^Long-term methods: intra-uterine device (IUD) and implants.

^4^ Permanent methods: male and female sterilization.

^5^The wealth variable is constructed at the household level (for each urban sample). Women are thus not evenly distributed in the three wealth groups.

^6^p-value based on Chi-square tests.

^7^Less than 3% of women in the sample had no education.

^8^The source of method is categorised as Public (n = 800), Private (n = 821) and Other (n = 95), with Other including NGOs.

Table 3 also shows that on average about 50% of all urban current users of modern contraceptives sought their method from a public source. The proportion using a private source for FP methods gradually rose from around 44% in 1993 to 55% in 2003, and then went down to 51% in 2008/09. Expectedly, there is a positive association between wealth and education and use of a private source of contraception. In particular, the urban rich are about twice as likely across the four survey periods, to resort to a private source, compared to the urban poor (e.g. 67.1% versus 31.7% in 2008/09). Likewise, urban women with secondary education or higher are more likely to use a private source, compared with their peers with no or primary education. As with the disparities by wealth, the magnitude of differences by educational levels remained largely unchanged over time.

### Pro-poor FP/RH initiatives in Kenya

The review of FP/RH programming in Kenya shows that a gradual focus on the poor, the marginalized, and hard-to-reach started to emerge from the late 1990s, in a context shaped by the Poverty Eradication Commission (established in 1999) and its wide range of mandates, including promotion of policies and pilot strategies for eradicating poverty, and mobilization and management of resources for direct poverty reduction activities with a strong focus on the poorest section of the population [32].

Guided by the realization that many public sector FP programs were unable to meet the growth in demand for services, and by the increasing unmet need for family planning among the poor and hard-to-reach groups, the United Sates Agency for International Development (USAID) – by far the largest funded of FP/RH in Kenya - pioneered in early 2000s the notion of “targeting” in contraceptive security planning, advocating for the concentration of scarce resources for the people most in need [33]. USAID’s POLICY Project (and its successor, the Health Policy Initiative) began to support FP/RH policy and advocacy in Kenya, mainly through its EQUITY Framework. This framework involved engaging and empowering the poor; quantifying the level of inequalities in healthcare access and health status; understanding the barriers to service access and use; integrating equity goals into policies, plans, and strategies; targeting resources and efforts to reach the poor; and yielding public-private partnerships for equity [34,35].

Prioritization of the poor, and particularly the urban poor continued to gain traction in Kenya in the early 2000s. The United Kingdom’s Department for International Development (DFID) - another major Kenya development funder - issued in 2001 a seminal position paper which stated that “the achievement of the international development targets will depend in part on the development of strategies which recognize the important role played by cities and towns in strengthening poor people’s capacity to improve their socio-economic and political condition” [36]. The USAID’s EQUITY Framework and DFID’s strategic orientation on better health for poor people guided the grant-making strategies of major FP/RH funding institutions covering Kenya. One such program is the voucher project launched by KfW (the German development bank) in 2005, in collaboration with the Kenyan government. Introduced in three rural districts and two urban slums, the project enables poor women to access highly subsidized safe motherhood and long-acting and permanent FP methods [37,38]. This increased focus of programs on the poor, including the urban poor, is likely correlated with the trend in poor-rich gaps in contraceptive use observed above in the quantitative findings.

## Summary and discussion

We have examined the differences in contraceptive use between the rich and poor in urban Kenya over time, to investigate the extent to which family planning services are increasingly reaching the urban poor. A clear finding from this analysis is the dramatic change in the patterns of contraceptive use between 2003 and 2008/09 that resulted in virtually no gap between the poor and the rich (as defined by household wealth) in 2008/09. The finding on the dwindling poor-rich inequities in the use of modern method of FP was confirmed, to a lesser extent by the analysis of the differences between women with primary education and women with secondary education (considered to be another dimension of poverty status). Results from a 2010 survey of about 11,000 households in urban Kenya confirm the insignificant difference in modern CPR between the poor (lowest wealth quintile) and the rich (highest quintile), with CPR among the rich not more than three percentage points higher than among the poor [39].

Our analysis focuses on trends in contraceptive use between the poor and rich over the last 15 years. The observed narrowing of the poor-rich gap in contraceptive use is programmatically important and likely reflects multiple factors including changes in the propensity to implement fertility desires through uptake of modern methods. We preferred to examine current use levels in this paper rather than unmet need as done in other studies since our focus was on actual gaps in use rather than potential use levels as measured by unmet need. We also sought to explore the extent to which the shrinking of the poor-rich gap in the use of modern contraceptive methods may be explained, at least partly, by an increased use of traditional methods by the rich or more educated women, with abortion as a possible back-up plan. As indicated earlier, use of traditional methods remained low and did not show any difference by wealth or education. Coupled with the finding that differentials in unintended pregnancy by wealth did not exhibit any variation over time, our main result on the narrowing gap in contraceptive use suggests a differential resort to abortion or method-choice between the poor and the rich.

## How do these results relate to the broader policy and program context?

In Kenya (and urban Kenya in particular), the use of modern contraceptive methods increased between 1993 and 1998 in the context of a strong commitment of the government and substantial funding and technical support from a range of bilateral and multilateral partners. Contraceptive use stalled during the following period, as a consequence of weakening prioritization of FP and RH in national and international policy agendas. Finally, contraceptive use increased markedly between 2003 and 2008/09 when the government’s efforts to recoup the lost decade became more aggressive [6,21,23,26]. Our findings indicate that while the overall improvement in the use of FP between 1993 and 1998 did not significantly benefit the urban poor, the recent positive trends carried a disproportionate increase among the urban poor. The evidence of narrowing poor-rich inequities in urban Kenya has also been documented for other health outcomes. A recent re-analysis of the Kenya DHS showed that among urban infants born in the five years preceding the 1993 survey, those from poor households were 72% more likely (poor-rich ratio of 1.72) to die before their first birthday, compared to their counterparts from rich households. The poor-rich gap widened in 1998 (ratio of 2.5), but went down noticeably in 2003 and 2008/09 (ratio of about 1.25 in both time periods). A similar trend was also observed for under-five mortality [7].

Judging by our review of the FP and RH program context in Kenya, the late 1990s marked the realization among the major development partners of the increasing unmet need for FP among the poor and hard-to-reach groups. This led to increased programs that deliberately targeted the poor - and particularly the urban poor – with FP and RH services [32-36]. Indeed, the specific vulnerability of the urban poor to adverse RH outcomes deserves attention because they constitute the fastest growing subpopulation in many sub-Saharan African countries [16].

Our findings on the type of contraceptive methods used show that not only do most urban women use short-term, less effective methods of contraception, but more importantly, the proportion of long-acting method users dropped by half between 1993 and 2008/09, from 39.0% to 18.2%. As expected, less privileged women (by wealth or education) are more likely to resort to short-term methods than their better-off peers. Given that the discontinuation and failure rates for short-term methods are typically higher than long-term methods [24], the heavy reliance on short-term methods may explain the discrepancy between the relatively high level of contraceptive use in urban Kenya, and the high level of unintended pregnancies and births. As noted by Magadi and Curtis [24], while short-term oral contraceptives and injectables constitute a viable option in contexts where spousal support is lacking, FP programs should encourage the promotion of male involvement initiatives, and ensure that women have the opportunity to make informed choices among the methods available. The development in 2008 of a national strategy to improve the uptake of long-acting and permanent methods of contraception in the FP program is undoubtedly a move in the right direction. The strategy seeks to equip health workers with knowledge and skills on LAPM provision; increase awareness, knowledge, and acceptability of LAPMs in the communities; increase funding and commitment for procurement of LAPM commodities; and strengthen public-private sector partnerships [40].

The role of the private sector in the provision of FP/RH services is being fostered in many FP programs. This study shows that in urban Kenya, the proportion of private sector users increased from 43.5% in 1993 to 55.1% in 2003, but declined to 50.8% in 2008/09. Across all survey periods, over 65% of rich urban women seek their method from the private sector, compared to less than 34% among the poor. To consolidate the progress towards reaching the urban poor, the Kenyan government should harness the potential of private clinics operating in urban, resource-deprived settings [41,42].

Before concluding, a few limitations of the trends analysis should be noted. First, the results are based on cross-sectional trends and thus it is not possible to demonstrate causality between programmatic activities and increased use among the urban poor. Second, there was a change in number and types (and perhaps the meaning) of assets measured in each survey year; therefore, the wealth index was constructed separately for each year and may have different interpretations over time. The wealth index used, however, is the standard approach used by program managers and policy makers in Kenya and elsewhere with DHS data as the primary data informing programs and policies. Likewise, the comparability of women’s education across time may be subject to question, as achieving primary education in 1993 may have a different implication on a woman’s life, compared to achieving the same level in 2008/09. Future analyses comparing the use of other methods like the relative index of inequality or the slope index of inequality to the wealth index used here, at least at the descriptive stage, would provide additional insights into the inequalities of contraceptive use by wealth, employment, education and other indicators over time.

## Conclusion

Despite a relatively high level of contraception use in Kenya, the levels of unplanned fertility remain disturbingly high, including in urban areas, presumably due to the overwhelming use of short-term methods. The narrowing gap in the recent past between the urban poor and the urban rich in the use of modern contraception is undoubtedly good news, which, coupled with the review of the family planning program context, suggests that FP programs are increasingly reaching the urban poor. Family planning programs should continue to focus on removing barriers to contraceptive uptake and scaling up the good practices identified in the FP literature [12,43]. Potential strategies could include the strengthening of the private sector as a key player in the provision of FP information and services, and the development of more effective linkages between the public and the private sectors to manage access to FP commodities and referrals [30]. By prioritizing the rapidly growing urban population, it is expected that FP programs’ effects will subsequently trickle out to rural areas leading to greater impacts overall [44].

## Competing interests

The authors declare that they have no competing interests.

## Authors’ contributions

JCF conceptualized the study, performed the analyses and wrote the first draft of the paper. ISS worked closely with JCF on paper conceptualization and paper writing. PK and VL led the review of population and family planning in Kenya. CM supported the review of FP/RH initiatives in Kenya. All authors reviewed and approved the final manuscript.
